# Inhibition of lysine acetyltransferases impairs tumor angiogenesis acting on both endothelial and tumor cells

**DOI:** 10.1186/s13046-020-01604-z

**Published:** 2020-06-05

**Authors:** Marta Di Martile, Chiara Gabellini, Marianna Desideri, Marta Matraxia, Valentina Farini, Elisabetta Valentini, Simone Carradori, Cristiana Ercolani, Simonetta Buglioni, Daniela Secci, Massimiliano Andreazzoli, Donatella Del Bufalo, Daniela Trisciuoglio

**Affiliations:** 1 Preclinical Models and New Therapeutic Agents Unit, Department of Research and Advanced Technologies, IRCCS Regina Elena National Cancer Institute, Rome, Italy; 2 Unit of Cell and Developmental Biology, Department of Biology, University of Pisa, Pisa, Italy; 3 Institute of Molecular Biology and Pathology, National Research Council, Rome, Italy; 4 Department of Pharmacy, University “G. d’Annunzio” of Chieti-Pescara, Chieti, Italy; 5 Pathology Unit, Department of Research and Advanced Technologies, IRCCS Regina Elena National Cancer Institute, Rome, Italy; 6 Department of Chemistry and Technologies of Drugs, “Sapienza” University, Rome, Italy

**Keywords:** Endothelial cells, Lung cancer cells, Tubulin, Acetylation

## Abstract

**Background:**

Understanding the signalling pathways involved in angiogenesis, and developing anti-angiogenic drugs are one of the major focuses on cancer research. Herein, we assessed the effect of CPTH6, a lysine acetyltransferase inhibitor and anti-tumoral compound, on angiogenesis-related properties of both endothelial and cancer cells.

**Methods:**

The in vitro effect of CPTH6 on protein acetylation and anti-angiogenic properties on endothelial and lung cancer cells was evaluated via wound healing, trans-well invasion and migration, tube formation, immunoblotting and immunofluorescence. Matrigel plug assay, zebrafish embryo and mouse xenograft models were used to evaluate in vivo anti-angiogenic effect of CPTH6.

**Results:**

CPTH6 impaired in vitro endothelial angiogenesis-related functions, and decreased the in vivo vascularization both in mice xenografts and zebrafish embryos. Mechanistically, CPTH6 reduced α-tubulin acetylation and induced accumulation of acetylated microtubules in the perinuclear region of endothelial cells. Interestingly, CPTH6 also affected the angiogenesis-related properties of lung cancer cells, and conditioned media derived from CPTH6-treated lung cancer cells impaired endothelial cells morphogenesis. CPTH6 also modulated the VEGF/VEGFR2 pathway, and reshaped cytoskeletal organization of lung cancer cells. Finally, anti-migratory effect of CPTH6, dependent on α-tubulin acetylation, was also demonstrated by genetic approaches in lung cancer cells.

**Conclusion:**

Overall, this study indicates that α-tubulin acetylation could play a role in the anti-angiogenic effect of CPTH6 and, more in general, it adds information to the role of histone acetyltransferases in tumor angiogenesis, and proposes the inhibition of these enzymes as an antiangiogenic therapy of cancer.

## Background

Angiogenesis is a crucial step in the transition of tumors from a benign state to a malignant one [1] and its inhibition is a promising tactic in limiting cancer progression [2]. Direct targeting of endothelial cells (ECs) may prevent the formation of new blood vessels, thus avoiding the limits of conventional cancer therapies [1, 3, 4]. Even if some anti-angiogenic agents are already tested in clinical trials [3, 4], current experimental data suggest that there are several adaptive mechanisms facilitating the resistance to these therapies [2, 3], including vasculogenic mimicry (VM), an alternative process that involves tumor vascularization primed by tumor cells [5].

Besides direct targeting of ECs and angiogenic factors, an alternative approach for cancer therapy involves modulation of epigenetic processes [6, 7], which include histone modifications. Acetylation of lysine residues, which is typically catalyzed by enzymes with histone acetyltransferase (HAT) or histone deacetylase (HDAC) activity, has become one of the most studied [8, 9]. While the HDACs can affect endothelial angiogenic functions by controlling specific genes/pathways involved in different phases of angiogenesis [10–14], the function of HATs in ECs biology and blood vessel development remains entirely unexplored. A recent study demonstrates that vascular endothelial growth factor receptor 2 (VEGFR2) undergoes ligand-dependent lysine acetylation, suggesting that acetylation is a critical mechanism that directly affects its function [15]. Moreover, reduction of HAT GCN5 represses bone marrow stromal cells-mediated angiogenesis [16]. In order to explore the hypothesis that HATs may provide key therapeutic targets for manipulation of angiogenic responses and new clues for anti-angiogenesis therapies, we investigated the effect of the HAT inhibitor 3-methylcyclopentylidene-[4-(4′-chlorophenyl)thiazol-2-yl] hydrazone (CPTH6) [17–19] on both ECs and cancer cells by using in vitro and in vivo functional assays.

## Materials and methods

### Cell cultures

Human commercially available established lung cancer lines (H1299, A549) were cultured in RPMI medium (Euroclone, Milan, IT) supplemented with 10% inactivated fetal bovine serum (HyClone, Thermoscientific, South Logan, UT), and antibiotics. The human umbilical vein endothelial cell line (HUVEC) was cultured in complete EBM medium (Lonza, Basel, Switzerland).

### Reagents preparation and treatment

3-Methylcyclopentylidene-[4-(4′-chlorophenyl)thiazol-2-yl] hydrazone (CPTH6), was dissolved in dimethyl sulfoxide (DMSO, Sigma-Aldrich, St. Louis, MO, USA) and diluted to the final concentrations in complete medium. As control, cells were treated with 1% DMSO. Zebrafish embryos at 6 h post-fertilization (hpf) or 24 hpf were immersed in E3 medium solutions containing CPTH6 at concentrations ranging from 1 to 20 μM for 24–120 h. As control, embryos were exposed to 0.1% DMSO. Where indicated, embryos at 48 hpf were injected in the perivitelline space in the proximity of developing subintestinal vein.

### In vitro and in vivo angiogenic assays and immunohistochemistry

For cell proliferation assay, HUVEC were seeded in 96-well culture plates (5 × 10^3^ cells/well), starved for 24 h and then treated with CPTH6. After 72 h, cells were processed as previously reported [20]. For migration and invasion assays, 5 × 10^4^ HUVEC were seeded into upper chamber of Transwell (Corning, Costar, New York, USA) or CultreCoat cell invasion chamber (Trevigen, Gaithersburg, MD, USA), respectively processing them after 6 h as previously reported [21]. For tube formation assay, 2.5 × 10^4^ HUVEC or 5 × 10^4^ lung cancer cells were seeded in duplicates in 24-well culture plates containing polymerized matrigel (BD Bioscience, San Jose, CA, USA) and incubated with CPTH6 for 6 or 18 h, respectively. HUVEC were also exposed for 6 h to conditioned medium (CM) obtained from H1299 cells treated for 24 h with the compound. Then the number of intersection points and branch length in ten random microscopic fields were counted. All procedures involving animals were authorized by the decree n. 26/2014 of the Italian Ministry of Health, authorization n. 787/2015PR-29/07/2015. In vivo angiogenesis matrigel assay was performed as previously reported [21]. Tumor vascularization of LCSC136 xenografts after treatment with CPTH6 [17] was evaluated by detecting CD31-positive cells in 5 μm paraffin sections by a streptavidin-biotin enhanced immunoperoxidase technique in an automated autostainer (BondTM Max, Leica BioSystem). For each tumor, three different 5 μm sections were analyzed and examined by light microscopy.

### Immunofluorescence and time-lapse microscopy

After CPTH6 treatment, cells seeded on coverslips were fixed and permeabilized before immunofluorescence. For analysis of F-actin filament, cells were incubated with phalloidin conjugated-rhodamine (Thermo Fisher Scientific). DNA was counterstained with 0.02 μg.ml^− 1^ DAPI (Sigma-Aldrich). Preparations were examined under an Olympus AX70 microscope using a 100X/1.35 NA objective. Images were acquired using a TCH-1.4ICE camera (Tucsen, Fujian, CHINA) controlled by ISCapture and processed by Photoshop CS. NIH ImageJ 1.3 software to quantify fluorescence intensity over the cell area from images acquired under identical exposure settings. For live microscopy experiments, cells were seeded at 80–90% confluency in μ-slides 4-well (Ibidi, Martinsried, DE), exposed to CPTH6 1 h later wound generation by 10 μl tip and time–lapse recording were conducted in a microscope stage incubator (Basic WJ, Okolab, Naples, IT) using an Eclipse Ti inverted microscope (Nikon, Tokyo, JA). Images were acquired over 48 h at 15 min intervals for phase contrast. Videos and still images were processed and analyzed using NIS-Elements AR 4.0. Cell migration ability was determined as previously described [22].

### Western blot analysis

Total protein extracts were fractionated by SDS-PAGE, transferred to a nitrocellulose filter and subjected to immunoblot assay (Supplementary Matherials). Antibody binding was visualized by chemiluminescence according to manufacturer’s specification and recorded on autoradiographic film (Amersham Biosciences).

### qRT-PCR, ChIP, and ELISA assays

Secreted VEGF level in CM was evaluated by ELISA assay (R&D Systems, Minneapolis, Minnesota, USA) by following manifacturer’s instructions and normalized to adherent cell number.

Total RNA was extracted using a Qiagen RNeasy Mini kit (Qiagen, Hilden, Germany) according to the manifacturer’s instructions. Reverse Transcription was performed using Reverse Transcription kit (Thermo Scientific) and GeneAmp PCR System 9700 (Applied Biosystems, Foster City, CA, USA). mRNA quantification was performed by SYBR Green-based qRT-PCR. All reactions were performed in triplicate using QuantStudio 6 Flex System (Applied Biosystems). The mRNA levels were normalized using β-actin. The following primers were used. VEGF Fw: TCTTCAAGCCATCCTTGTTG, Rv: TCTGCATGGTGATGTTGGAC; VEGFR1 Fw: TGGCTGCGACTCTCTTCTG, Rv: CAAAGGAACTTCATCTGGGTCC; VEGFR2 Fw: TGGGGGAGCGTGTCAGAAT, Rv: CCGCTTTAATTGTGTGATTGGAC; β-actin Fw: ATTGCCGACAGGATGCAGAA, Rv: GCTGATCCACATCTGCTGGAA. The results were evaluated by the 2^-ΔΔCt^.

For ChIP assay, 2 × 10^6^ H1299 were plated onto 150 mm dish and, after 24 h, treated with CPTH6. After 24 h cells chromatin was crosslinked with formaldhyeide 1% and fragmented through sonication. Chromatin was immunoprecipitated overnight with anti-acetyl histone H3 (Millipore, Billerica, MA, USA) or RNA Polymerase II (Pol II, Abcam, Cambridge, UK) antibodies. A 1400 bp region in the VEGF promoter upstream the transcription initiation site and containing consensus sites recognized by several transcription factors were analysed by amplifying three regions: P1 (between -54 bp and -290 bp); P2 (between -512 bp and -757 bp) and P3 (between -1147 bp and -1391 bp). The following primers were used: P1 Fw: TGCTGCATTCCCATTCTCAGT, Rv: ATCTTCCCTAAGTGCTCCCAAAG; P2 Fw: AGACTCCACAGTGCATACGTG, Rv: AGTGTGTCCCTCTGACAATG; P3 Fw: CTTCGAGAGTGAGGACGTGTGT, Rv: GGAGCAGGAAAGTGAGGTTACG. Quantization of immunoprecipitated DNA was performed in triplicate on the QuantStudio 6 Flex System using SYBR Green Detection method. The results were evaluated by the 2^-ΔΔCt^.

### Zebrafish care

Animal procedures were performed following protocols approved by Italian Ministry of Health and local Ethical Committee of the University of Pisa (authorization n. 99/2012-A, 19.04.2012), in conformity with Directive 2010/63/EU. Transparent Casper embryos (*roy*^−/−^;*nacre*^−/−^) [23] and transgenic zebrafish Tg (*kdrl*: EGFP; *gata1*:dsRed) embryos, expressing GFP and dsRed under the control of ECs and erythroid lineage specific promoters, respectively [24], were obtained by natural mating and maintained at 28 °C in E3 medium.

### Whole-mount alkaline phosphatase vessel staining

Zebrafish larvae at 72 hpf were fixed in 4% paraformaldehyde, dehydrated with ethanol/PBST series and stained for endogenous alkaline phosphatase activity as previously described [25] using NBT/BCIP staining solution (Roche Diagnostics GmbH, Penzberg, Germany). Images were acquired using a stereomicroscope Nikon SMZ1500.

### Statistics

Experiments were replicated three times, unless otherwise indicated, and data were expressed as average ± standard deviation (SD). Differences between groups were analysed with a two-sided paired or unpaired t-test, one-way ANOVA followed by a post hoc Tukey test or Chi Square analysis and were considered to be statistically significant for *p* < 0.05. Mann-Whitney test was used for correlation studies.

## Results

### CPTH6 impairs in vivo and in vitro angiogenesis

**Fig. 1:**
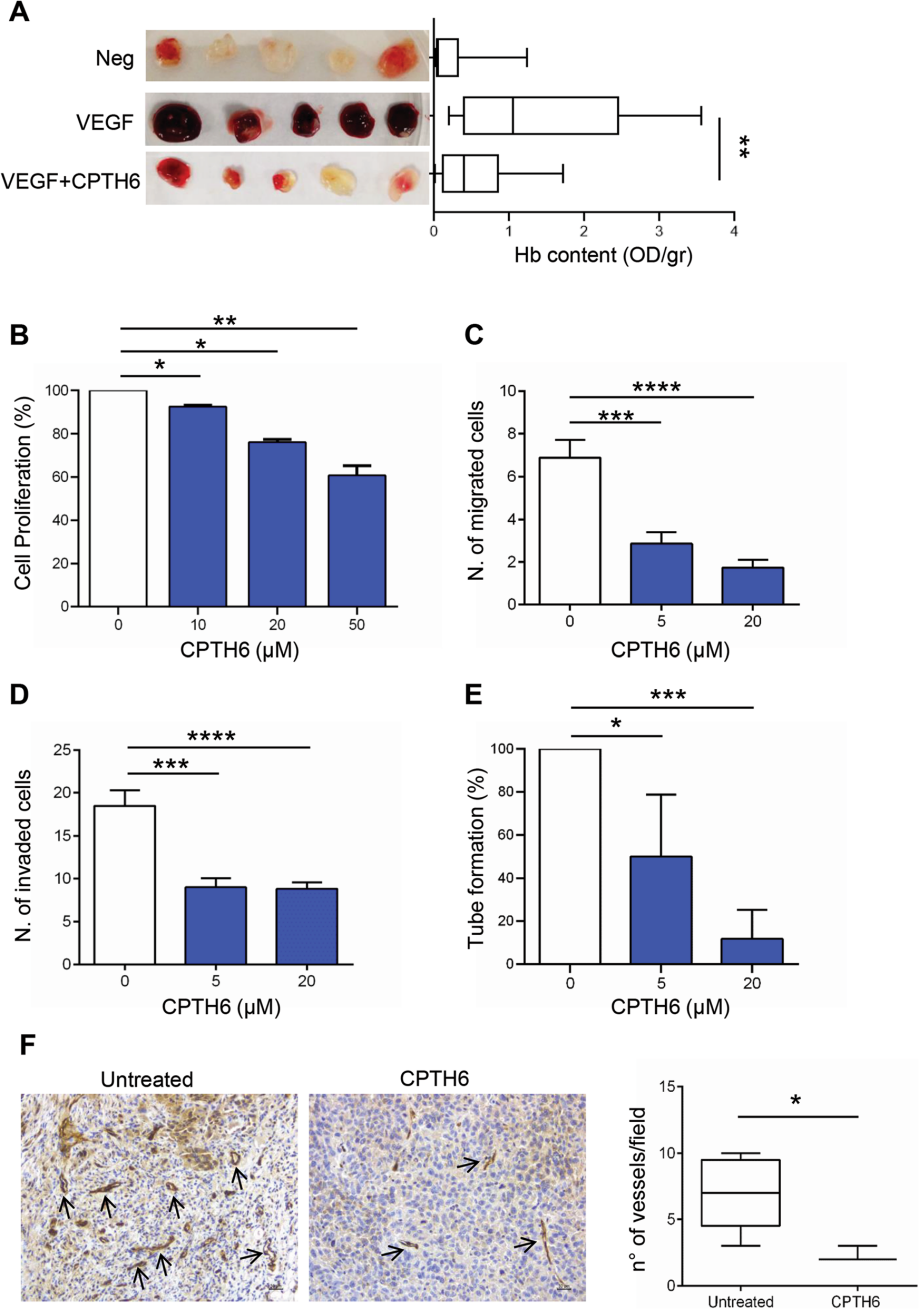
**a** Representative images of matrigel plugs and hemoglobin content of in vivo vessel formation assessed after injection of C57BL/6 mice with matrigel plugs containing PBS (Negative control), VEGF alone (Positive control) or VEGF in combination with 50 μM CPTH6. Five matrigel plugs/group of a representative experiment are shown. The values were expressed as optical density (OD)/gr matrigel plug. *p*-values were calculated between positive and CPTH6-containing matrigel plugs. **b** Analysis of proliferation of HUVEC exposed to increasing concentrations of CPTH6 for 72 h. The results are reported as percentage of “proliferation of CPTH6-treated cells/proliferation of control cells”. The results represent the average ± SD of three independent experiments. **c**,**d** Quantification of in vitro cell migration (**c**) and cell invasion (**d**) of HUVEC exposed to the indicated concentrations of CPTH6 for 6 h. The results are reported as average ± SD of number of migrated or invaded cells. **e** Quantification of capillary-like structure formation in HUVEC exposed to the indicated concentrations of CPTH6 for 6 h. The results are reported as percentage of tube formation relative to control, and represent the average ± SD of three independent experiments. **f** Representative images and relative quantification of immunohistochemical detection of microvessel density by CD31 staining in LCSC136 tumors from control or CPTH6-treated mice. NOD/SCID mice inoculated subcutaneously with 2.5 × 10^5^ LCSC136 cells and treated intraperitoneally with CPTH6 (50 mg/Kg; 5 days every 24 h for 3 weeks), IHC analysis was performed at the end of treatment. Magnification 20X. Scale bar, 30 μm. **b-f***p*-values were calculated between control and treated cells. *p < 0.05; **p < 0.01; ***p < 0.001; ****p < 0.0001.

In order to study the possible effect on angiogenesis of CPTH6, a thiazole derivative inducing HAT inhibition [17, 19], we performed matrigel plug angiogenesis assay in mice. As shown in Fig. 1a, matrigel plugs containing VEGF showed a stronger angiogenic response if compared with negative PBS-containing plugs. Interestingly, addition of CPTH6 disrupted VEGF-induced neovascularization. Indeed, Hemoglobin (Hb) content was significantly reduced by about 50% in presence of CPTH6 if compared to positive controls. These data led us to test CPTH6 effects on angiogenic features of HUVEC. As shown in Fig. 1b and S1A, CPTH6 reduction of cell proliferation is associated to weak accumulation of cells in the G_0_/G_1_ cell-cycle phase and to less than 2.5% accumulation in subG_1_ phase.

We also analyzed CPTH6 effect on HUVEC migration, invasion, and differentiation, which represent key events in the angiogenic process. As shown in Fig. 1c and S1B, HUVEC ability to migrate in response to a chemoattractant dropped of about 60 and 75% in presence of 5 μM and 20 μM CPTH6, respectively, when compared to control cells. Similarly, HUVEC ability to invade was reduced by about 50% in presence of 5 μM and 20 μM CPTH6, when compared to control cells (Fig. 1d and S1C). Moreover, CPTH6 severely impaired HUVEC tube formation in a dose-dependent manner (Fig. 1e and S1D).

Next, we evaluated the effect of CPTH6 on in vivo tumor vascularization of patient-derived LCSC136 xenografts. As shown in Fig. 1f, tumor xenograft sections from CPTH6-treated tumors showed a reduced CD31-positivity when compared to control xenografts, suggesting that the anti-tumor activity of CPTH6 may rely also on its ability to affect tumor vascularization.

**Fig. 2:**
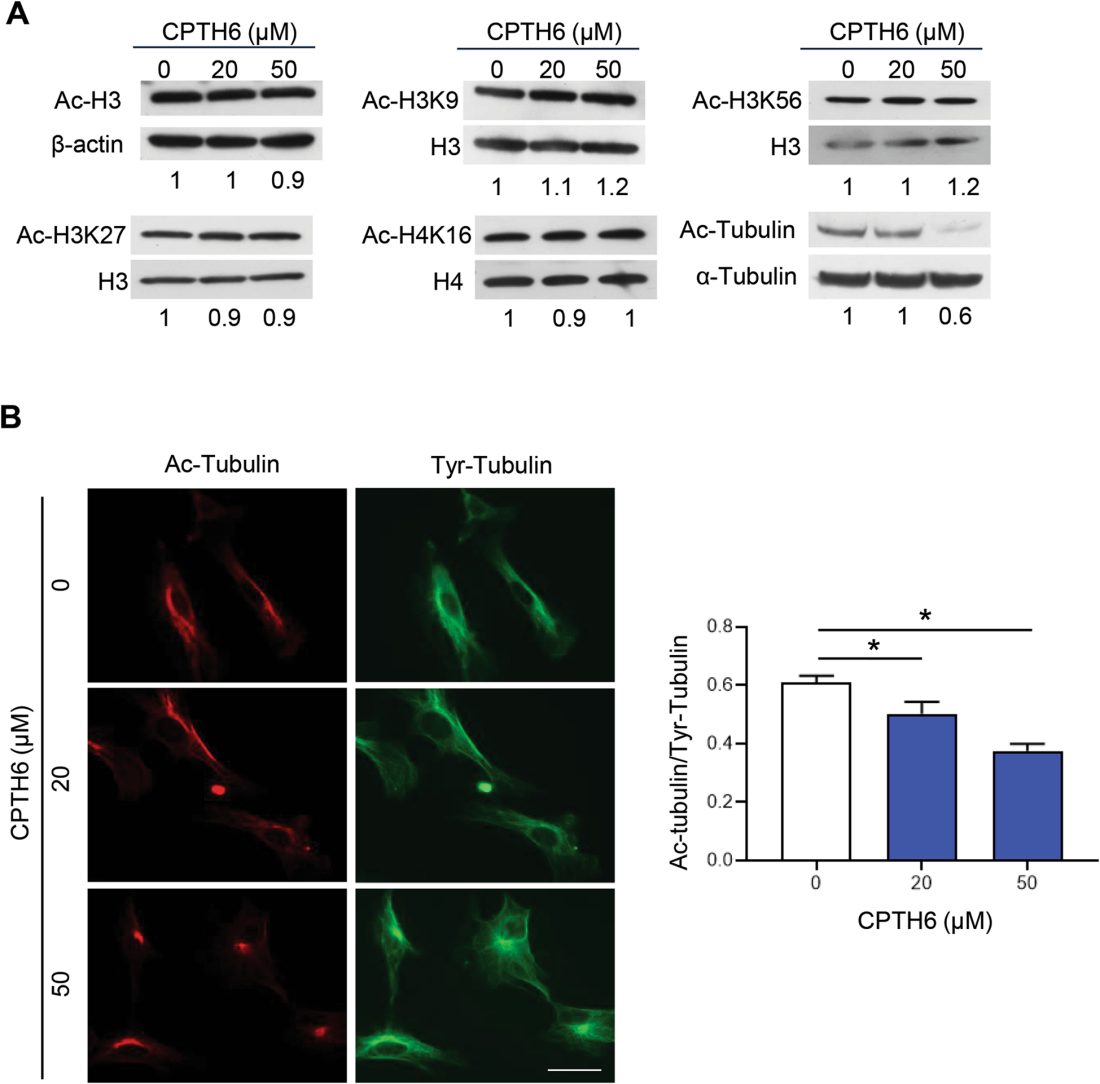
**a** Western blot analysis and relative quantification by densitometric analysis of total or acetylated histone H3 pan (Ac-H3) or at K9 (Ac-H3K9), K27 (Ac-H3K27), K56 (Ac-H3K56), and histone H4 at K16 (Ac-H4K16) and α-tubulin at K40 (Ac-tubulin) in extract of HUVEC exposed to indicated concentrations of CPTH6 for 72 h. Values are expressed as fold change of acetylated proteins relative to total ones. **b** Immunofluorescence images (left) and relative quantification (right) of tyrosinated (Tyr) and acetylated (Ac) α-tubulin in HUVEC exposed to increasing concentrations of CPTH6 for 72 h. p-values were calculated between control and treated cells. *p < 0.05. Magnification 63X. Scale bar, 50μm

We previously reported that CPTH6 treatment reduces histone acetylation in a cell type–dependent manner [19]. CPTH6 did not affect histone H3 pan-acetylation in HUVEC neither H3K9 acetylation, as well as H3K27, H3K56 and H4K16 (Fig. 2a). Of note, CPTH6 induced a remarkable reduction of the cytosolic protein α-tubulin acetylation at K40 residue in HUVEC (Fig. 2a) as previously demonstrated in cancer cell lines [17–19]. These data were confirmed by immunostaining of acetylated and tyrosinated α-tubulin forms, the latter was used as a control. Notably, acetylated and tyrosinated microtubules in control cells are well distributed between the peripheral and perinuclear region (Fig. 2b). Upon CPTH6 treatment, acetylated microtubules are concentrated in perinuclear region whilst a reduction of acetylated microtubules at the periphery of cells is well evident. On the contrary, no change in the localization or intensity of tyrosinated microtubules was observed in CPTH6-treated cells. Collectively, these results indicate that CPTH6 inhibits in vitro α-tubulin acetylation and reduces in vitro endothelial angiogenesis-related functions.

### CPTH6 affects the VEGF/VEGFR2 pathway of lung cancer cells

**Fig. 3:**
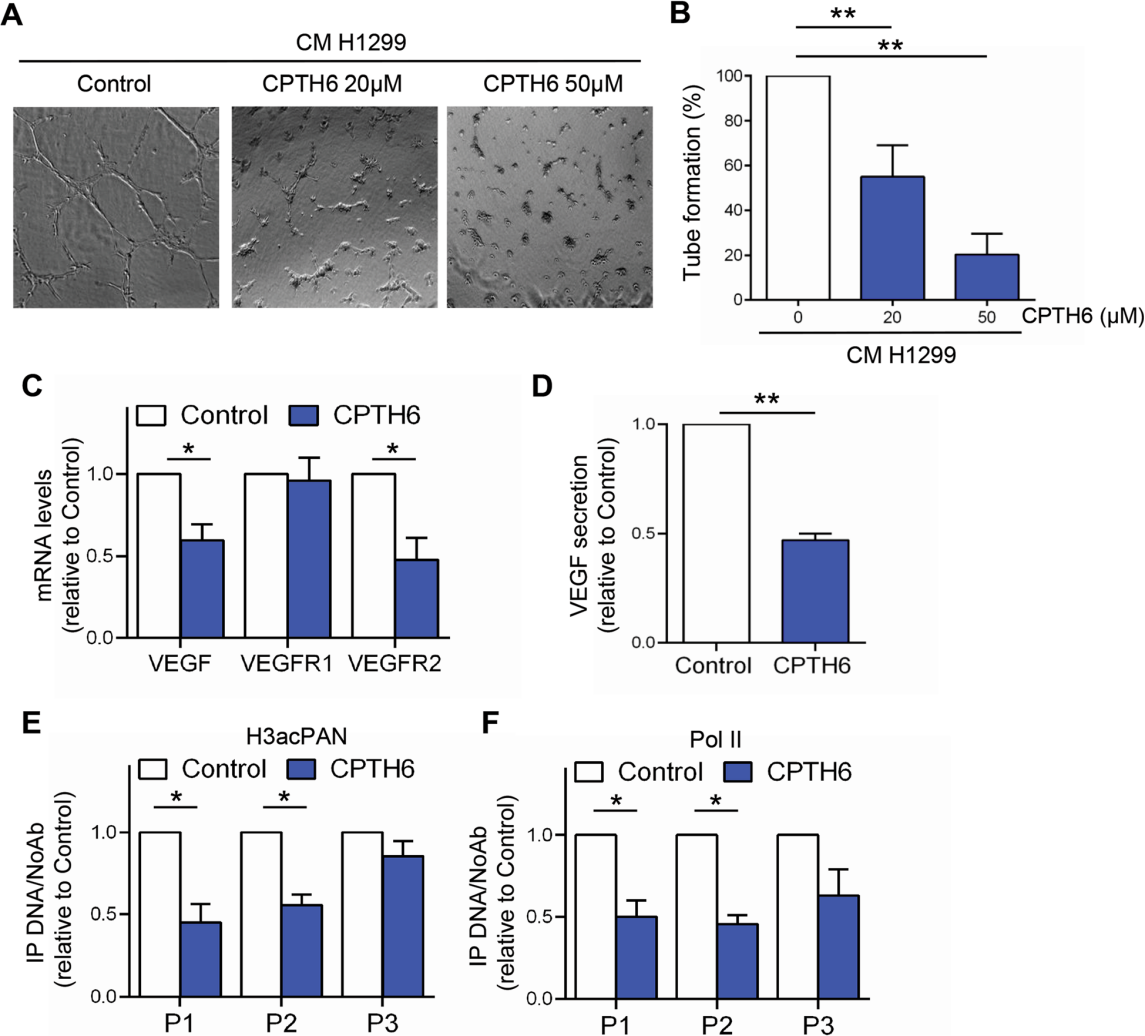
**a** Representative images and **b** relative quantification of morphogenesis assay of HUVEC plated on matrigel exposed for 6 h to the conditioned media (CM) from control or H1299 cells treated with CPTH6 for 24 h at the indicated concentrations. **c** Analysis of VEGF, VEGFR1 and VEGFR2 mRNA expression in H1299 cells exposed to 20 μM CPTH6 for 24 h. **d** Quantification of VEGF protein secretion performed by ELISA assay in CM from H1299 control cells or treated as reported in (**c**). **e**,**f** ChIP analysis of (**e**) histone H3 acetylation (H3acPAN) and (**f**) Pol II recruitment at three regions (P1-P3) of VEGF promoter of H1299 control cells or treated as in (**c**). The results represent the average ± SD (**b,d**) or ± SEM (**c**,**e**,**f**) of three independent experiments. *p* values were calculated between control and treated conditioned medium (**a**,**b**,**d**) or between control and treated cells (**c**,**e**,**f**). *p < 0.05. **p < 0.01

We next explored whether CPTH6 might also modulate angiogenesis acting indirectly on ECs through its effect on cancer cells. To this aim, HUVEC were exposed to CM from control or CPTH6-treated H1299 lung cancer cells, and their ability to organize a capillary network was assessed. When exposed to CM from H1299 control cells, HUVEC formed tube-like structures resembling a capillary plexus. Conversely, partially organized and rounded ECs were observed after the addition of CM from CPTH6-treated cells (Fig. 3a, b).

We next explored the possible effect of CPTH6 on the expression of VEGF, an angiogenic molecule that modulates angiogenesis of cancer cells and ECs through tyrosine kinase VEGF receptors (VEGFRs). Thus, VEGF, VEGFR1 and VEGFR2 expression upon CPTH6 treatment was studied by qRT-PCR in H1299 cells (Fig. 3c). Interestingly, we found that CPTH6 reduced the mRNA expression of VEGF and VEGFR2 while it did not affect the expression of VEGFR1. Consistent with these data, ELISA assay confirmed VEGF protein downregulation by CPTH6 treatment in CM from lung cancer cells (Fig. 3d). To further investigate the effect of CPTH6 on VEGF transcription, ChIP was used to determine whether CPTH6 treatment specifically affected H3 acetylation on VEGF promoter. Interestingly, CPTH6 treatment resulted in a reduction of H3 acetylation in two out of three sites analysed. In particular, the H3 acetylation was reduced of about 50% in P1 and P2 regions after CPTH6 treatment, whereas the P3 region, was not affected (Fig. 3e). In accord, CPTH6 treatment significantly reduced the Pol II recruitment at the P1 and P2 regions (Fig. 3f). Notably, no modulation of VEGF and VEGFRs expression has been found in ECs upon exposure to CPTH6 (Figure S2A).

### CPTH6 impairs the organization of vascular-like structures and cell migration of lung cancer cell line

**Fig. 4:**
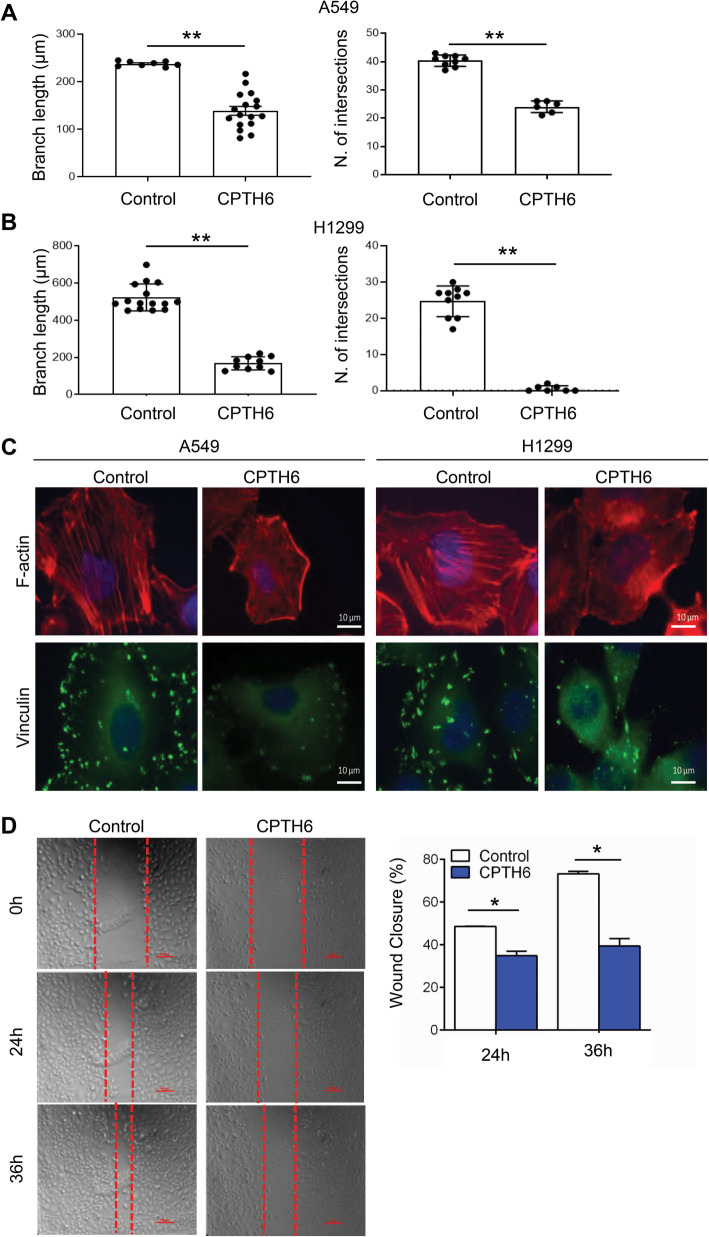
**a**,**b** Quantification (branch length and number of intersection points) of capillary-like structure formation in A549 (**a**) and H1299 (**b**) cells plated on matrigel and treated with 20 μM CPTH6 for 18 h. The results represent the average ± SD of two independent experiments. **c** Representative images of immunofluorescence conducted in A549 and H1299 cells control or treated with 50 μM CPTH6 for 24 h using rhodamine-conjugated phalloidin to visualize F-actin fibers or for vinculin expression and localization. Blue fluorescence represents DAPI stained nuclei. **d** Representative images and relative quantification of migration of A549 cells control or treated with 50 μM CPTH6 for 48 h and subjected to time-lapse videorecording. The images were recorded every 15 min, and were taken at the starting point (0 h), after 24 and 36 h. The red outlines show the gap area. The migration rates of two different conditions (control and CPTH6) were determined as the percentage of wound closure or the percentage of area reduction. Magnification 10X. Scale bar, 100 μm. **a**, **b**, **d** p values were calculated between control and CPTH6-treated cells. *p < 0.05; **p < 0.01

VM normally evolves in highly invasive tumors as an alternative way to form a vascular network and may represent a compensative mechanism by which tumor cells counteract the antiangiogenic therapy [26]. When plated on matrigel, both A549 (Fig. 4a) and H1299 (Fig. 4b) cells exhibited ability to organize themselves in capillary-like structures, indicative of VM, but CPTH6 impaired this process, as demonstrated by the significant decrease of both tubules intersections and length (Fig. 4a,b and S2B). Notably, this effect of CPTH6 on VM is not due to apoptosis induction (data not shown). As VM is a process in which tumor cells develop highly patterned channel structures via rearrangement of F-actin cytoskeleton and matrix remodelling, we analysed whether CPTH6 might affect cytoskeleton organization and cell motility. Similarly to what observed in HUVEC, CPTH6 treatment reduced acetylated microtubules localized at cell periphery of both A549 and H1299 (Figure S2C). As illustrated in Fig. 4c, in control cells F-actin fibers appear very distinct, dense and aligned, while CPTH6 determined a loss of F-actin fibers. Indeed, CPTH6 causes an accumulation of broken, misaligned actin fibers in both cell lines. Notably, CPTH6 also caused a significant reduction in the average number of focal adhesions per cell, from 39 ± 6 to 20 ± 6 in A549 line while the 65 ± 23 focal adhesions/cell in H1299 control cells are reduced to 13 ± 6 by CPTH6 treatment. Additionally, CPTH6 treatment determined a significant reduction of A549 cells migration rate, expressed as the percentage of wound closure (Fig. 4d).

**Fig. 5:**
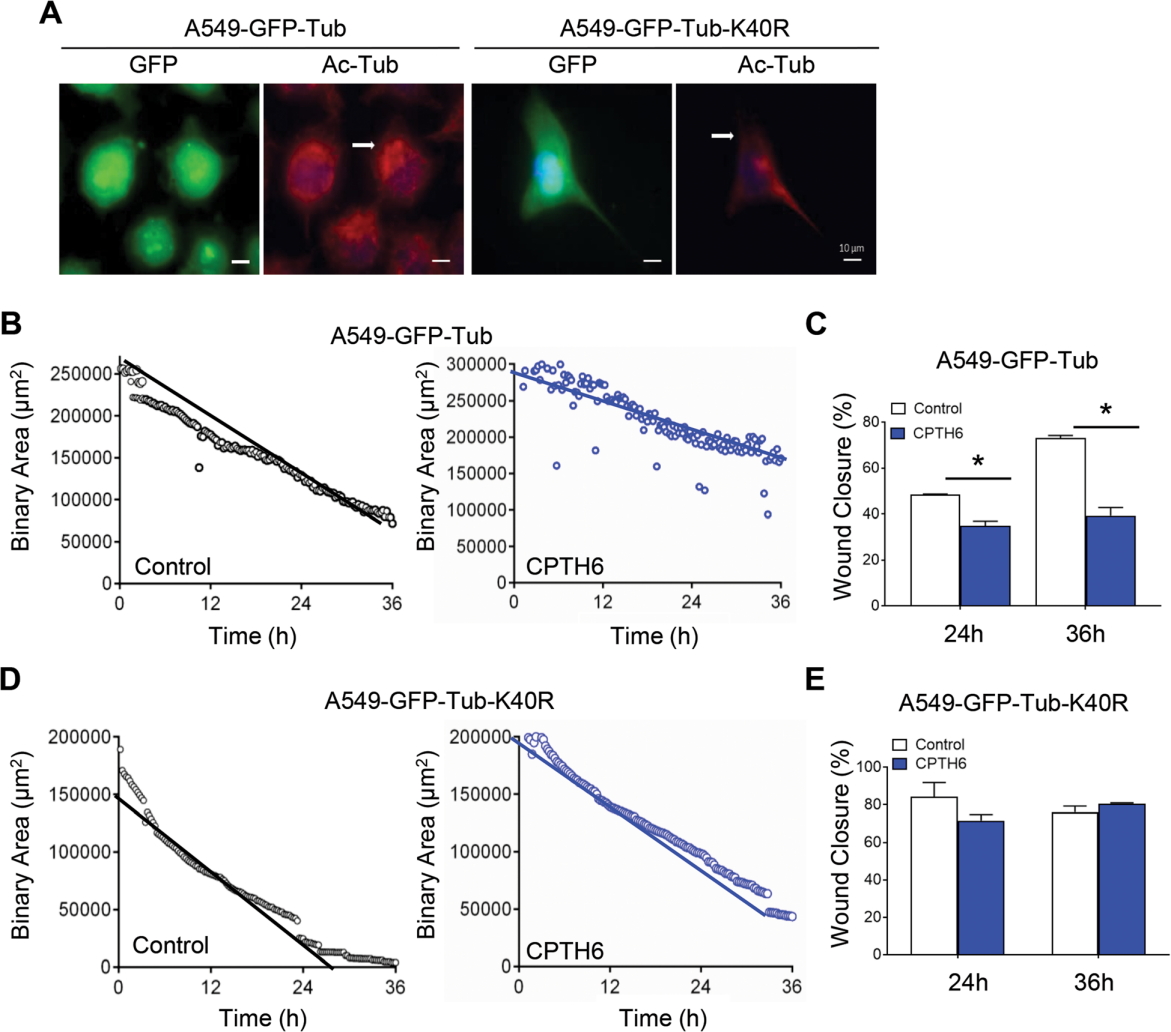
**a** Representative images of immunofluorescence for DNA (blue), GFP (green), and acetylated α-tubulin (red) of A549 cells overexpressing α-tubulin WT-GFP (A549-GFP-Tub) or α-tubulin K40R-GFP (A549-GFP-Tub-K40R). Scale bar, 10 μm. **b**-**e** Quantification of migration rate of A549 cells (**b**) overexpressing α-tubulin WT-GFP, and (**d**) α-tubulin K40R-GFP treated with CPTH6 for 48 h and subjected to time-lapse videorecording using a 10X objective. The sheet migration rates of two different conditions (control and CPTH6-treated) were determined by plotting the binary area versus time. **c**,**e** The migration rates of A549 cells (**c**) overexpressing α-tubulin WT-GFP and (**e**) α-tubulin K40R-GFP in response to CPTH6 were determined as the percentage of wound closure. p values were calculated between control and treated cells. *p < 0.05

As CPTH6 antitumoral effect correlates with the baseline level of acetylated α-tubulin in lung cancer cells [17], we hypothesized that α-tubulin acetylation might be also implicated in the effect of CPTH6 on cell motility. To determine the impact of α-tubulin acetylation on CPTH6-induced lung cell migration impairment, we transfected cells with α-tubulin wild type (WT)-GFP or mutant K40R α-tubulin-GFP (Fig. 5a). As depicted in Fig. 5b,c, CPTH6 treatment on α-tubulin WT-GFP-overexpressing cells resulted in decreased migration speed and gap closure. Conversely, K40R α-tubulin-GFP overexpression has a strong impact on the effect of CPTH6 on A549 cell migration by attenuating the anti-migratory effect of CPTH6 (Fig. 5d,e), suggesting that the anti-migratory effects of CPTH6 on lung cancer cells depend on its effect on α-tubulin acetylation.

### CPTH6 affects vessel development in zebrafish

**Fig. 6:**
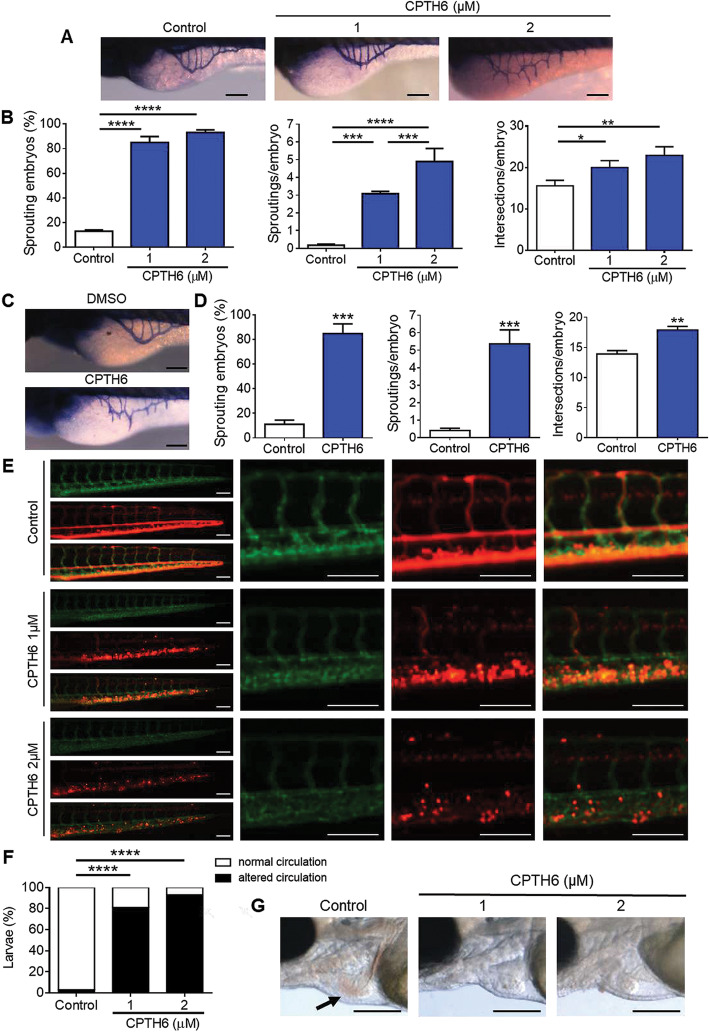
**a** Representative images and **b** quantification of subintestinal veins (SIVs) development alteration, evidenced by whole-mount alkaline phosphatase staining in Casper zebrafish embryos at 72 hpf exposed to 1 and 2 μM CPTH6 at 6 hpf. **c** Representative images and (**d**) quantification of SIVs development alteration evidenced by whole-mount alkaline phosphatase staining in Casper zebrafish embryos at 72 hpf injected in the yolk with 50 μM CPTH6 solution at 48 hpf. **b**,**d** Development alteration was evaluated in terms of sprouting embryos (100%), sproutings/embryo, intersections/embryo. The error bar represents the standard error of the mean (SEM) of 3 independent experiments (*N* = 75 larvae/experiment). **e** Representative images of GFP-positive ISVs development and Dsred-positive erythroid cells and **f** percentage of larvae with altered circulation in Tg (kdrl: GFP;gata1:dsRed) Casper zebrafish at 72 hpf exposed to 1 and 2 μM CPTH6 at 6 hpf. At higher magnification the accumulation of gata1-positive erythroid cells is evidenced in caudal erythropoietic tissue of CPTH6-treated embryos. **f** A representative experiment of 4 independent experiments (*n* = 30–40 larvae/experiment) with the same result is shown. **g** Bright-field micrographs of Casper zebrafish larvae at 96 hpf exposed to 1 and 2 μM CPTH6 at 6 hpf. Arrow indicates the normal color of blood. Scale bar, 200 μm. **a**-**g** As control, zebrafish larvae were exposed to DMSO solution. **b**,**d**,**f** *p < 0.05, **p < 0.01, ***p < 0.001, ****p < 0.0001. (**a**, **c**, **e**) Scale bar, 100 μm

Having shown that CPTH6 treatment decreases in vivo angiogenesis in a mouse model, we extended our analysis to the in vivo model of teleost zebrafish. Embryos at early gastrulation stage (6 hpf) were exposed to increasing concentrations of CPTH6 for 24, 48 and 72 h. As shown in Figure S3A, CPTH6 determined a dose-dependent reduction of embryo survival while the time of exposure did not have an impact in modulating CPTH6-induced embryo mortality, thus identifying the first 24 h of treatment as the most critical for embryo survival. For this reason, embryos at 24 hpf were exposed to CPTH6 ranging from 1 to 20 μM, demonstrating that neither 24 h nor 48 h of CPTH6 treatment have an impact on zebrafish embryo survival (Figure S3B). Interestingly, the exposure of zebrafish embryos to CPTH6 determined an alteration of cardiac development, indicated by the increase of looping angle in embryos at 48 hpf (Figure S3C-F) along with head size reduction, mirroring the GCN5/pCAF knockdown phenotype [27]. In order to analyze the effect of CPTH6 on angiogenesis, we evaluated the effect CPTH6 on subintestinal veins (SIVs) formation at 72 hpf. As evidenced in Fig. 6a, 1 μM CPTH6 induced the appearance of anomalous sprouting vessels, while a deep alteration of vessel development, characterized by thinner blood vessels, was observed in embryos exposed at 2 μM CPTH6 when compared to control embryos. Indeed, CPTH6 treatment induced an increase in the percentage of embryos characterized by sprouting vessels, a dose-dependent increase of number of sproutings per embryo and an increase of the number of intersections per embryo when compared to control embryos (Fig. 6b). Since the normal vessel plexus from SIVs develops between 48 and 72 hpf, we investigated the effect on angiogenesis of acute exposure to a high-dose of CPTH6 by injecting embryos with 50 μM CPTH6 in the perivitelline space at 48 hpf. After 24 h, SIVs vessel development is strongly impaired by CPTH6 (Fig. 6c,d). Along with SIVs, intersegmental vessels (ISVs) of the trunk have usually been used to monitor angiogenesis during zebrafish development. Using the reporter zebrafish line expressing GFP and dsRed under the control of ECs and erythroid lineage-specific promoters, respectively [24], we observed an alteration of ISVs development occurring in zebrafish embryos exposed to CPTH6, as shown in Fig. 6e. In particular, there was a reduction of the number of vessels through which a circulation of gata1-positive erythroid cells was observed, especially in larvae exposed to 2 μM CPTH6 (Fig. 6f). The alteration of vessel development in CPTH6-treated larvae, probably due to an anastomosis of ISVs, was associated to a general reduction of circulating erythroid cells and an increased accumulation in the posterior caudal vein plexus/caudal hematopoietic tissue (Fig. 6e), which is the site of hematopoietic stem cells expansion and differentiation [28]. The possible alteration of erythropoiesis induced by CPTH6 exposure was also supported by the observation of blood circulation through the heart of larvae at 96 hpf. As evidenced in Fig. 6g, CPTH6-treated larvae were characterized by a reduced blood circulation with a change in blood color from red to pale/transparent, thus suggesting a reduced level of Hb in erythroid cells. Interestingly, we also observed a delayed hatching and reduced locomotor activity in larvae exposed to CPTH6 (Figure S4).

## Discussion

In this study CPTH6 was shown to inhibit in vitro endothelial cell functions, as well as in vivo neo-vascularization. CPTH6 inhibits the angiogenic process through inhibition of in vitro cell migration, invasion, and ability to assembly the vascular network. In line with the cell context-dependent effect of CPTH6 ability to reduce histone acetylation in cancer cells [17–19], CPTH6 did not affect either histone H3/H4 acetylation or VEGF/VEGFRs expression of HUVEC. Given that CPTH6 may elicit its inhibitory activity on HATs by preventing the binding of acetyl-CoA [19], we can speculate that the context-dependent effect of CPTH6 could be due to the different metabolic properties of endothelial and tumor cells [29–31].

Furthermore, CPTH6 caused a remarkable reduction of α-tubulin acetylation on which the anti-angiogenic effect of CPTH6 on ECs could rely, independently from histone acetylation modulation effect on gene transcription. Besides reducing VEGF-induced angiogenesis in an in vivo matrigel assay performed in mice, CPTH6 exposure also alters vascular development in zebrafish embryos. In particular, we observed blood vessels thinning in CPTH6-exposed zebrafish larvae, paralleled by an evident reduction of blood circulation, thus indicating that the increased sprouting observed in these larvae leads to the generation of non-functional vessels. This effect of CPTH6 on zebrafish vascular early development may be due to an alteration of ECs functions, as suggested also by the accumulation of erythroid cells in the caudal hematopoietic tissue. In this stem cell niche, ECs are involved in a dynamic interplay with hematopoietic stems, which is crucial for their amplification and final differentiation [32]. Therefore, we speculate that CPTH6 could affect this ECs feature.

In addition to its direct effect on ECs, CPTH6 also impairs the interplay between endothelial and lung cancer cells. In particular, we demonstrated that CPTH6 treatment is associated with a decreased vascularization in xenografts from H1299 bearing mice. Moreover, CM derived from CPTH6-treated lung cancer cells impaired HUVEC ability to organize capillary-like network. These results can be explained by the evidence that CPTH6 reduced VEGF protein release and VEGF/VEGFR2 mRNA expression in H1299 cancer cells.

We also demonstrated that CPTH6 effectively impairs VM, by reducing the ability of lung cancer cells to form capillary-like structures. Notably, this effect is not due to apoptosis induction, but might be due, at least in part, to the ability of CPTH6 to affect autophagy [18]. In fact several evidences reported a strong link between VM formation and autophagy [33–35].

Interestingly, CPTH6 also reduced in vivo tumor vascularization of patient-derived LCSC136 xenografts.

The findings reported in this study clearly demonstrate the ability of CPTH6 to act on different cell players as ECs and tumor cells, thus indicating that the anti-tumor activity of CPTH6 may rely also on its ability to affect tumor vascularization. Further investigations are required to evaluate if CPTH6 may also elicit its functions acting on lymphatic endothelial cells or other important components of tumor niche as cancer-associated fibroblasts, tumor-associated macrophages, or other immune cells.

Another important finding of this study is the ability of CPTH6 to induce i) a reduction of acetylated microtubules at the periphery of the cells, ii) the disruption of F-actin stress fibres, iii) a reduction of focal adhesion, thus suggesting that the effect of CPTH6 on tubule formation may be mediated by an alteration of the cellular cytoskeleton architecture. This effect could be ascribed primarily to the reduction of K40-acetylated α-tubulin level induced by CPTH6 treatment.

α-tubulin acetylation levels are regulated in humans by opposing activities of the enzymes α-tubulin acetyltransferase 1 (ATAT1) and HDAC6. Despite other HAT enzymes are able to acetylate α-tubulin, ATAT1 is the only one that can acetylate α-tubulin at K40 in mammalian cells [36]. The role of α-tubulin acetylation in regulating cell motility and polarity is deeply debated and it appears to be dependent on the cellular context. In non-trasformed cells, HDAC6 overexpression mediated α-tubulin deacetylation and increases cell motility [37], and α-tubulin acetylation is required for cell–cell contact inhibition and cell adhesion [38]. Conversely, ATAT1 deficiency leads to reduced α-tubulin acetylation and impaired migration of cortical neurons [39]. Indeed, neuronal α-tubulin acetylation levels are reduced in ATAT1 knock-down zebrafish which are characterized by a strong alteration of embryo development and a reduced response to tactile stimuli [36].

α-tubulin acetylation was shown to regulate metastasis–associated properties in different tumor histotypes. In breast cancer, acetylated tubulin levels correlate with an enhanced metastatic behavior [40]. In lung cancer, ATAT1 plays a key role in cell migration and invasion through the modulation of epithelial–mesenchymal transition and cell polarity [41]. In colon cancer, ATAT1 downregulation inhibits cell proliferation and cell invasion through modulation of Wnt1/β-catenin signaling [42]. Our results indicate that the anti-migratory effects of CPTH6 on lung cancer cells are dependent of its effect on α-tubulin acetylation, thus suggesting that this compound may also inhibit ATAT1, in addition to GNC5 and pCAF. However, we cannot rule out that CPTH6 may also elicit its function acting on other substrates or affecting acetylation of other proteins contributing to the organization of the actin cytoskeleton and cell migration, as cortactin, or acting as key player in angiogenic cascade, as VEGFR2. Both these proteins are acetylated by CBP and/or p300 [15, 43, 44] but we exclude that CPTH6 may elicit its function inhibiting these HAT enzymes [19].

## Conclusion

Overall, the results of this study demonstrate that CPTH6, in addition to its antitumor properties, also plays a role in remodeling the cytoskeleton and in regulating cell migration and neo-angiogenesis and it adds information on the impact of altered α-tubulin acetylation statuson tumor progression.

## Supplementary information

**Additional file 1.**

**Additional file 2.**

## Data Availability

Materials are available upon request.
