# Supplementary material for: Inhibition of lysine acetyltransferases impairs tumor angiogenesis acting on both endothelial and tumor cells
Source: J Exp Clin Cancer Res. 2020 Jun 5;39:103. doi: 10.1186/s13046-020-01604-z (PMC7273677; doi:10.1186/s13046-020-01604-z)
Supplement: Supplementary file 1 — Additional file 1. [file 13046_2020_1604_MOESM1_ESM.pdf]

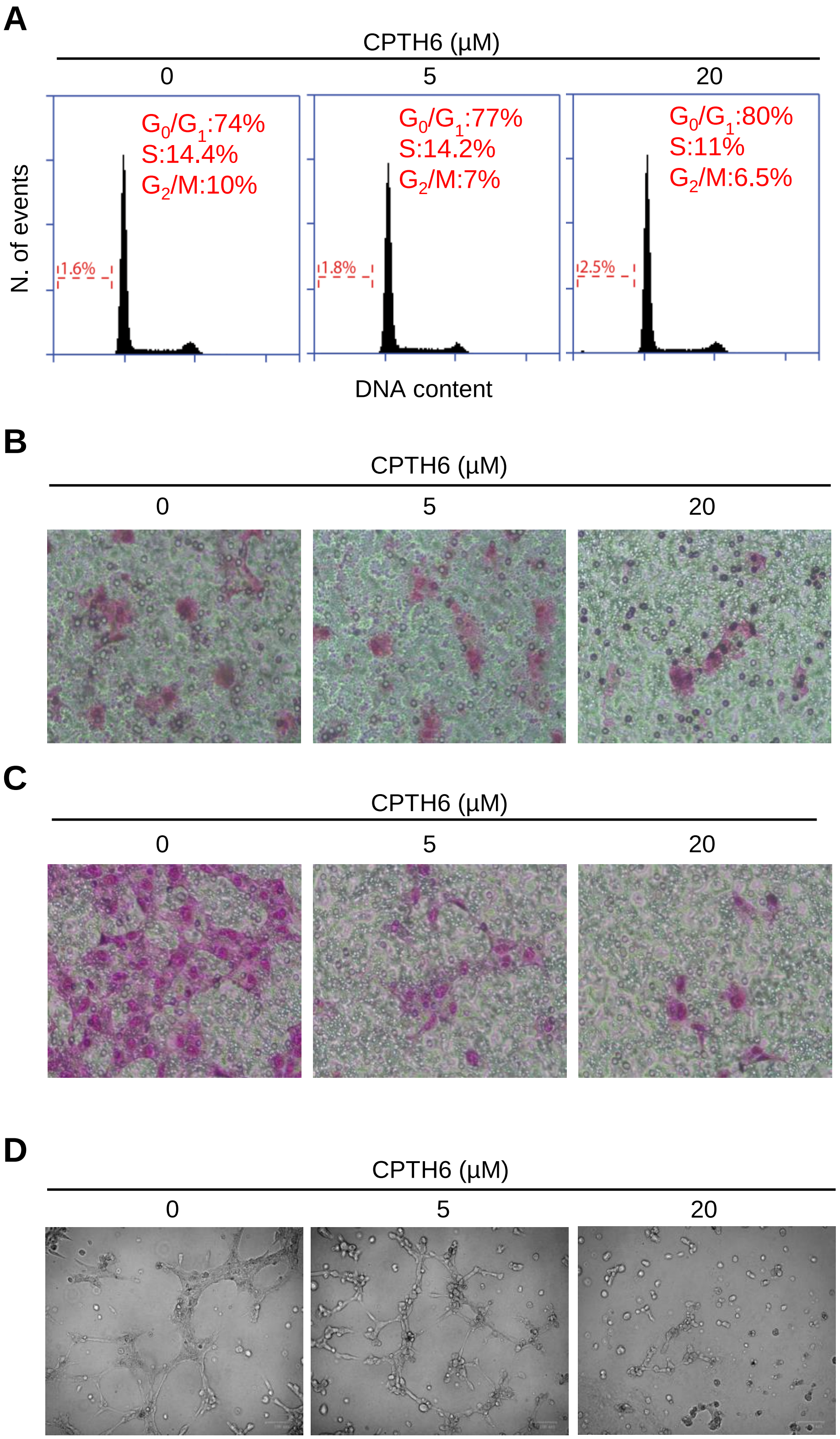

Figure S1. (A) Flow cytometric analysis of cell distribution and sub-G1 peak of HUVEC exposed to the indicated concentrations of CPTH6 for 72 h. Representative images of (B) migration, (C) invasion, (D) tube formation of HUVEC exposed to the indicated concentrations of CPTH6 for 6 h.

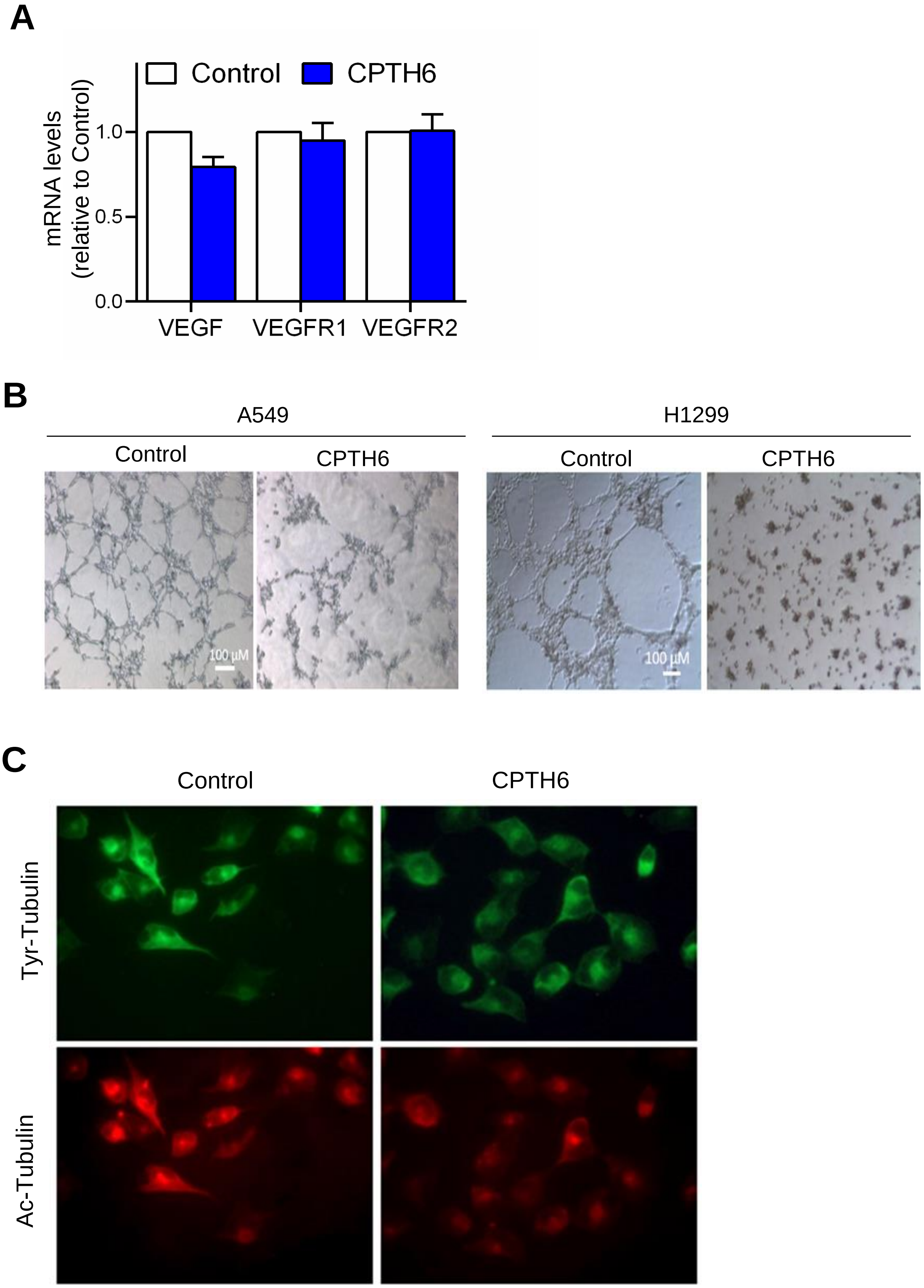

Figure S2. (A) qRT-PCR analysis of VEGF, VEGFR1 and VEGFR2 mRNA levels in HUVEC cells exposed to 20 µM CPTH6 for 24 h. The results represent the average ± SEM of three indipendent experiments. (B) Representative images of capillary-like structure formation in A549 and H1299 cells plated on matrigel with 20 µM CPTH6 for 18 h. (C) Immunofluorescence staining of H1299 lung cancer control cells or treated with 50 µM CPTH6 for 48 h with antibodies against α-tubulin, acetylated α-tubulin (Ac-Tubulin) or tyrosinated tubulin (Tyr-Tubulin) to visualize microtubules.

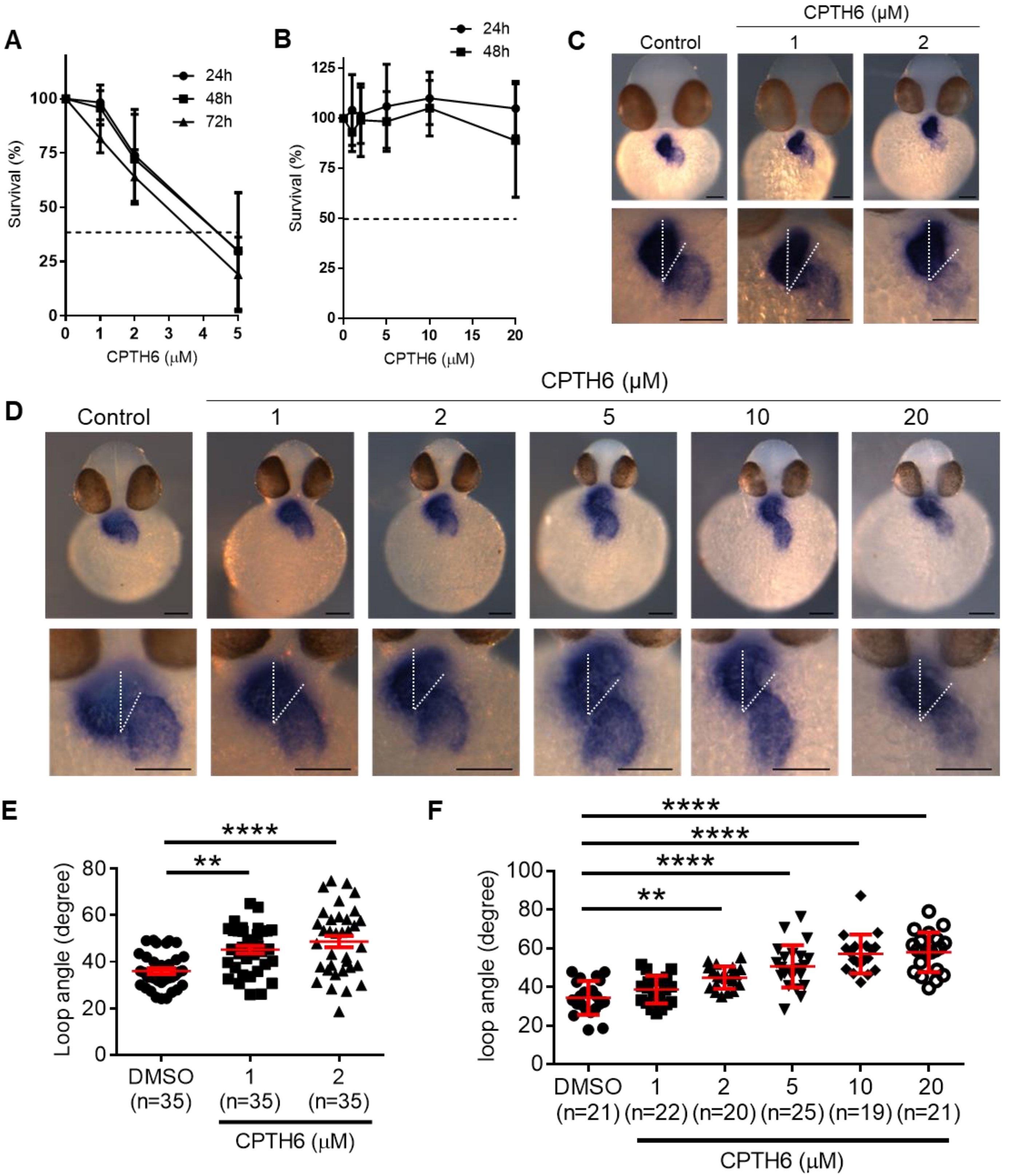

Figure S3. (A,B) Survival analysis of Casper zebrafish embryos exposed at (A) 6hpf or (B) 24hpf to increasing concentrations of CPTH6 for 24, 48 and 72h of treatment. The error bar represents the standard error of the mean (SEM) of 5 independent experiments (N=40 larvae/ experiment). (C,D) Representative images of in situ mRNA hybridization for cmlc2 and (E,F) average looping angle in Casper zebrafish embryos at 48 hpf (n=35) exposed to 1 and 2 μM CPTH6 at (C,E) 6hpf or (D,F) 24 hpf. The "looping angle" was defined as the angle created between the plane of the cardiac atrioventricular junction and the embryo anteroposterior axis, as indicated in the lower panels in (C,D). (A-F) As control, zebrafish larvae were exposed to DMSO solution. (E,F) The statistical significance of differences among groups was evaluated by one-way ANOVA, followed by a post hoc Tukey test. (F) The statistical significance of differences among DMSO and CPTH6 10μM was evaluated by Mann-Whitney test. **$p < 0.01$, ****$p < 0.0001$.

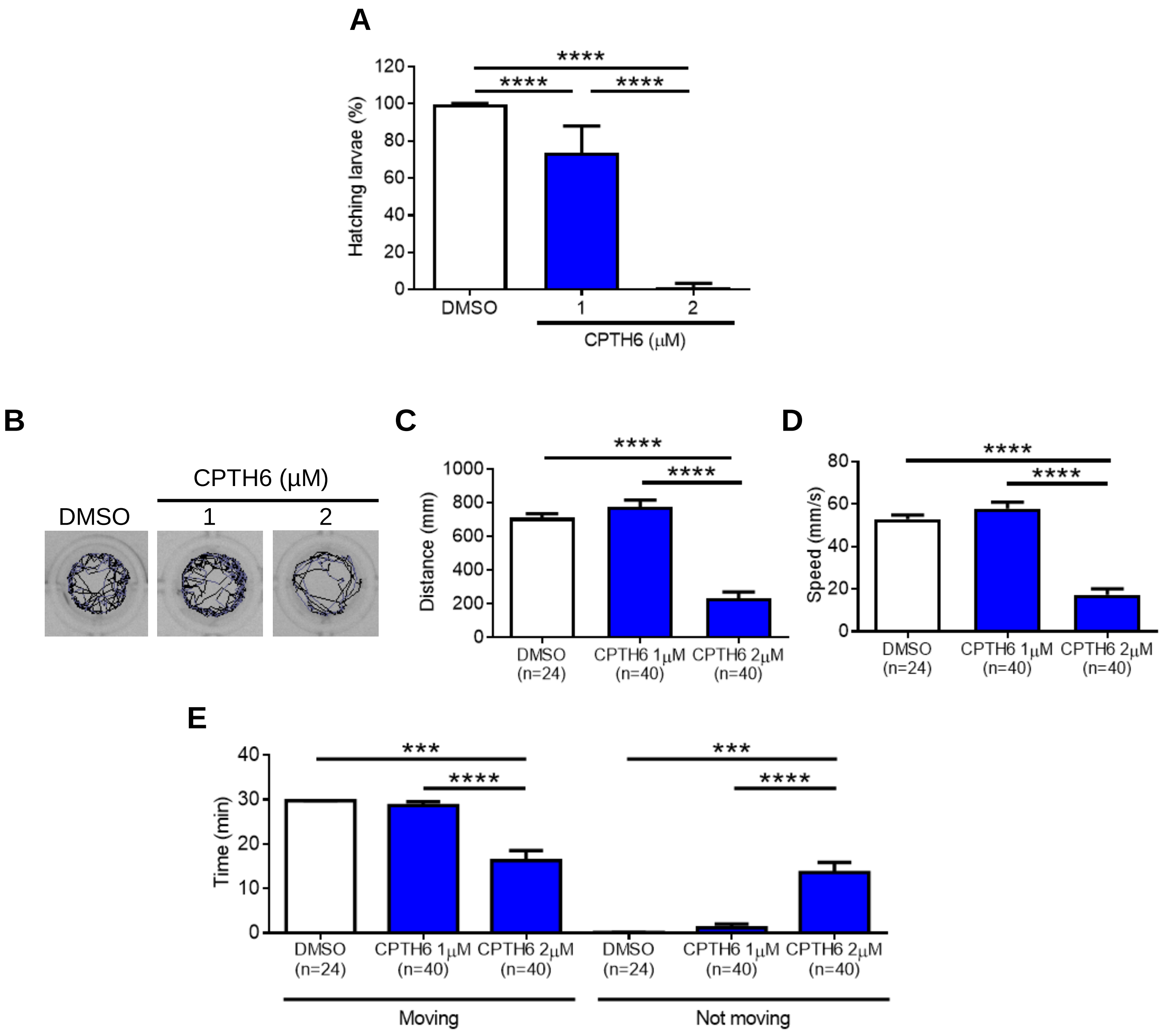

Figure S4. (A) Quantification of hatching in Casper zebrafish larvae at 72 hpf exposed to 1 and 2 μM CPTH6 at 6hpf. The error bar represents the standard error of the mean (SEM) of 10 independent experiments (N=40-50 larvae/experiment). (B) Representative images of locomotion tracking and quantification of (C) distance, (D) speed and (E) time spent moving or not moving in Casper zebrafish embryos at 120 hpf exposed to 1 and 2μM CPTH6 at 6hpf. (A-E) As control, zebrafish larvae were exposed to DMSO solution. (A) The statistical significance of differences among groups was evaluated by 1-way ANOVA, followed by a post hoc Tukey test. (C-E) The statistical significance of differences among groups was evaluated by Kruskal-Wallis test. ***$p < 0.001$; ****$p < 0.0001$.
