# Supplementary material for: Inhibition of lysine acetyltransferases impairs tumor angiogenesis acting on both endothelial and tumor cells
Source: J Exp Clin Cancer Res. 2020 Jun 5;39:103. doi: 10.1186/s13046-020-01604-z (PMC7273677; doi:10.1186/s13046-020-01604-z)
Supplement: Supplementary file 2 — Additional file 2. [file 13046_2020_1604_MOESM2_ESM.pdf]

Supplementary Matherials

Antibodies

Primary antibodies used for immunofluorescence and immunoblot experiments

**Antigen**	**Company**	**Clone**
CD31	Dianova GmbH, Warburgstr., Hamburg, Germany	SZ31
Acetyl-α-tubulin	Sigma-Aldrich, St. Louis, MO	6-11B-1
Tyrosyl-α-tubulin	Sigma-Aldrich, St. Louis, MO	YL1/2
vinculin	Santa Cruz	7F9
acetylated histone H3	Millipore, Billerica, MA, USA	6.6.2
acetyl-H3 (K9)	Cell Signaling, Freiburg, Germany	C5B11
acetyl-H3 (K27)	Cell Signaling, Freiburg, Germany	D5E4
acetyl-H3 (K56)	Cell Signaling, Freiburg, Germany	4243
acetyl-H4 (K16)	Cell Signaling, Freiburg, Germany	E2B8W
total H4	Cell Signaling, Freiburg, Germany	D2X4V
α-tubulin	Santa Cruz Biotechnology, Santa Cruz, CA, USA	DM1A
β-actin	Sigma-Aldrich, St. Louis, MO	AC15

Anti-mouse or anti-rabbit immunoglobulin G (IgG)-Secondary antibodies conjugated with horseradish peroxidase were purchased by Cell Signaling, Freiburg, Germany

Whole-mount in situ hybridization

Whole mount *in situ* hybridization was performed as previously described [1] with few modifications. Antisense RNA probe for myocardium-specific marker *cmlc2* (myl7, myosin light chain 7), cloned in pGEM-T easy vector, was transcribed *in vitro* using Sp6 RNA polymerase and digoxigenin (DIG) RNA labeling Mix (Roche Diagnostics Mannheim, Germany). Zebrafish embryos at 48 hpf were fixed with 4% PFA dehydrated with methanol/PBST series digested with proteinase K (Sigma) solution and incubated first in hybridization buffer containing 300 ng/ml DIG-labeled antisense RNA probes at 68 °C ON and then with anti-DIG Fab fragment conjugated with alkaline phosphatase (1:2000, Roche Diagnostics)followed by exposure to BM purple solution (Roche Diagnostics) and monitored closely for staining progression. Images were acquired using a stereomicroscope Nikon SMZ1500. The "looping angle", defined as the angle created between the plane of the cardiac atrioventricular junction and the embryo anteroposterior axis [2], was quantified using ImageJ software.

Zebrafish larvae locomotor activity

After the exposure to CPTH6 starting from 6 hpf, Casper embryos at 120 hpf were tracked for 30 min in a 96-well plate by DanioVision automated device (Noldus, Wageningen, Netherlands) and analyzed by Ethovision software (Noldus).

Supplementary references

[1] M. Andreazzoli, V. Broccoli, and I. B. Dawid, Cloning and expression of noz1, a zebrafish zinc finger gene related to drosophila nocA, Mech. Dev. 104 (2001) 117-120.

[2] Y. Chernyavskaya, A. M. Ebert, E. Milligan, and D. M. Garrity, Voltage-gated calcium channel CACNB2 (beta2.1) protein is required in the heart for control of cell proliferation and heart tube integrity, Dev. Dyn. 241 (2012) 648-662.
